# Impact of Exercise and Moderate Hypoxia on Glycemic Regulation and Substrate Oxidation Pattern

**DOI:** 10.1371/journal.pone.0108629

**Published:** 2014-10-16

**Authors:** Takuma Morishima, Ayaka Mori, Hiroto Sasaki, Kazushige Goto

**Affiliations:** 1 Graduate School of Sport and Health Science, Ritsumeikan University, Kusatsu, Shiga, Japan; 2 Faculty of Sport and Health Science, Ritsumeikan University, Kusatsu, Shiga, Japan; Tokyo Institute of Technology, Japan

## Abstract

**Objective:**

We examined metabolic and endocrine responses during rest and exercise in moderate hypoxia over a 7.5 h time courses during daytime.

**Methods:**

Eight sedentary, overweight men (28.6±0.8 kg/m^2^) completed four experimental trials: a rest trial in normoxia (FiO_2_ = 20.9%, NOR-Rest), an exercise trial in normoxia (NOR-Ex), a rest trial in hypoxia (FiO_2_ = 15.0%, HYP-Rest), and an exercise trial in hypoxia (HYP-Ex). Experimental trials were performed from 8:00 to 15:30 in an environmental chamber. Blood and respiratory gas samples were collected over 7.5 h. In the exercise trials, subjects performed 30 min of pedaling exercise at 60% of VO_2_max at 8:00, 10:30, and 13:00, and rested during the remaining period in each environment. Standard meals were provided at 8:30, 11:00, and 13:30.

**Results:**

The areas under the curves for blood glucose and serum insulin concentrations over 7.5 h did not differ among the four trials. At baseline, %carbohydrate contribution was significantly higher in the hypoxic trials than in the normoxic trials (*P*<0.05). Although exercise promoted carbohydrate oxidation in the NOR-Ex and HYP-Ex trials, %carbohydrate contribution during each exercise and post-exercise period were significantly higher in the HYP-Ex trial than in the NOR-Ex trial (*P*<0.05).

**Conclusion:**

Three sessions of 30 min exercise (60% of VO_2_max) in moderate hypoxia over 7.5 h did not attenuate postprandial glucose and insulin responses in young, overweight men. However, carbohydrate oxidation was significantly enhanced when the exercise was conducted in moderate hypoxia.

## Introduction

Obesity leads to multiple metabolic disorders, including insulin resistance and postprandial hyperglycemia. Exercise is an important ‘therapy’ in the treatment of postprandial hyperglycemia because muscle contraction stimulates glucose uptake by skeletal muscle [1]–[3]. Furthermore, we revealed that 4 weeks of endurance training under hypoxic conditions resulted in greater improvement of postprandial hyperglycemia than training under normoxic conditions [4]. In a study by Mackenzie et al., 2011 [5], type 2 diabetic patients rested or exercised for 60 min under hypoxic conditions. Blood glucose concentrations were significantly lower after 60 min of rest or exercise under hypoxic condition compared with baseline values. Although the mechanism of glucose lowering by hypoxic exposure is not fully understood, changes in substrate utilization patterns may be involved because acute exercise under hypoxic conditions increases carbohydrate oxidation [6]–[8]. In particular, enhanced glucose uptake by skeletal muscle is thought to account for increased carbohydrate oxidation under hypoxic conditions. Several studies have demonstrated that severe hypoxia stimulates the translocation of glucose across the plasma membrane [9], [10].

Kelly et al., 2010 [11] reported that plasma glucose response to a 75 g glucose load was significantly attenuated under severely hypoxic condition (a simulated altitude of 4300 m) in healthy adults. In an animal study, increased glucose uptake by the soleus muscle was observed after 1 week of exposure to severe (FiO_2_ = 10.0%) hypoxia [12]. Thus, severe hypoxic stimulation (a simulated altitude of> 4000 m) probably has a beneficial effect in preventing postprandial hyperglycemia through promoting glucose uptake by skeletal muscle. However, because the use of severe hypoxia would not be appropriate, due to the risk of acute mountain sickness (e.g., headache, nausea, and anorexia), exposure to *moderate* hypoxia (a simulated altitude of <3000 m) is more practical in terms of application for obese and overweight people. Although several studies reported that greater improvement of glucose tolerance by chronic staying or hiking at moderate or severe (altitude of 2200–4000 m) altitude was found [13]–[15], information about the influence of acute exposure to moderate hypoxia on glycemic regulation is still limited. Furthermore, no reported study has investigated the effect of rest and exercise under moderately hypoxic conditions on metabolic responses over a day in overweight subjects.

Given this context, the purpose of the present study was to examine the effects of rest and exercise under 7.5 h of moderately hypoxic conditions on substrate utilization and postprandial metabolic responses in overweight men. We hypothesized that the combination of rest and exercise under moderately hypoxic conditions would increase carbohydrate oxidation and attenuate postprandial blood glucose response over the day.

## Methods

### Subjects

Eight sedentary, overweight men participated. Their ages, physical characteristics, and fitness levels are presented in Table 1. The subjects were not participating in any training programs at the start of this study. All subjects were informed about the purpose of the present study and experimental procedure, and provided written informed consent. The study was approved by the Ethics Committee for Human Experiments at Ritsumeikan University, Japan.

**Table 1:** Physical characteristics and fitness.

Characteristics			
Age (yrs)		27±3	
Height (cm)		174.6±2.1	
Body weight (kg)		87±1	
BMI (kg/m2)		28.6±0.8	
Percentage fat (%)		28.4±2	
Waist hip ratio		0.92±0.1	
Waist circumference (cm)		97.0±2.1	
VO_2_max/BW (ml/min/kg)		37.9±1.9	

mean ± SE.

### Experimental design

Subjects visited the laboratory six times throughout the experimental period. During the first and second visits the subject's maximal oxygen uptake (VO_2_max) was assessed using a graded power test on an ergometer (828E, Monark, Stockholm, Sweden) under normoxic (first) and hypoxic (second) conditions. These tests were conducted on different days at least a week before experimental trial. The first load began at 60 W, and the load was increased progressively in 30-W increments every 2 min until exhaustion. The test was terminated when the subject failed to maintain the prescribed pedaling frequency of 60 rpm or reached an oxygen consumption (VO_2_) plateau. Respiratory gases were collected and analyzed using an automatic gas analyzer (AE300S, Minato Medical Science Co., Ltd, Tokyo, Japan). The data collected were averaged every 30 s. HR was measured continuously during the test using a wireless HR monitor (Acculex Plus; Polar Electro Oy). The test to measure VO_2_max was repeated under hypoxic conditions (FiO_2_ = 15.0%) on a separate day to determine the workload during an exercise trial under hypoxic conditions.

Four experimental trials were subsequently carried out in a randomized crossover design. Each trial was separated by ≥ 7 days. The experimental trials consisted of four different scenarios: a rest trial under normoxic condition (FiO_2_ = 20.9%, NOR-Rest), an exercise trial under normoxic condition (FiO_2_ = 20.9%, NOR-Ex), a rest trial under hypoxic conditions (FiO_2_ = 15.0%, HYP-Rest), and an exercise trial under hypoxic conditions (FiO_2_ = 15.0%, HYP-Ex). These four trials were selected to determine the influence of the independent effect of “moderate hypoxic exposure only” or “rest and exercise under moderately hypoxic conditions” on metabolic responses over a day. We selected 15.0% (a simulated altitude of 2700 m) of FiO_2_ as a “moderate hypoxia” because previous studies reported that endurance training under this level of hypoxia further improved glycemic regulation compared with the same training under normoxic conditions [4], [16]. All trials were completed in an environmental chamber. The temperature and relative humidity in the environmental chamber were maintained at 24°C and 40%, respectively.

**Figure 1:**
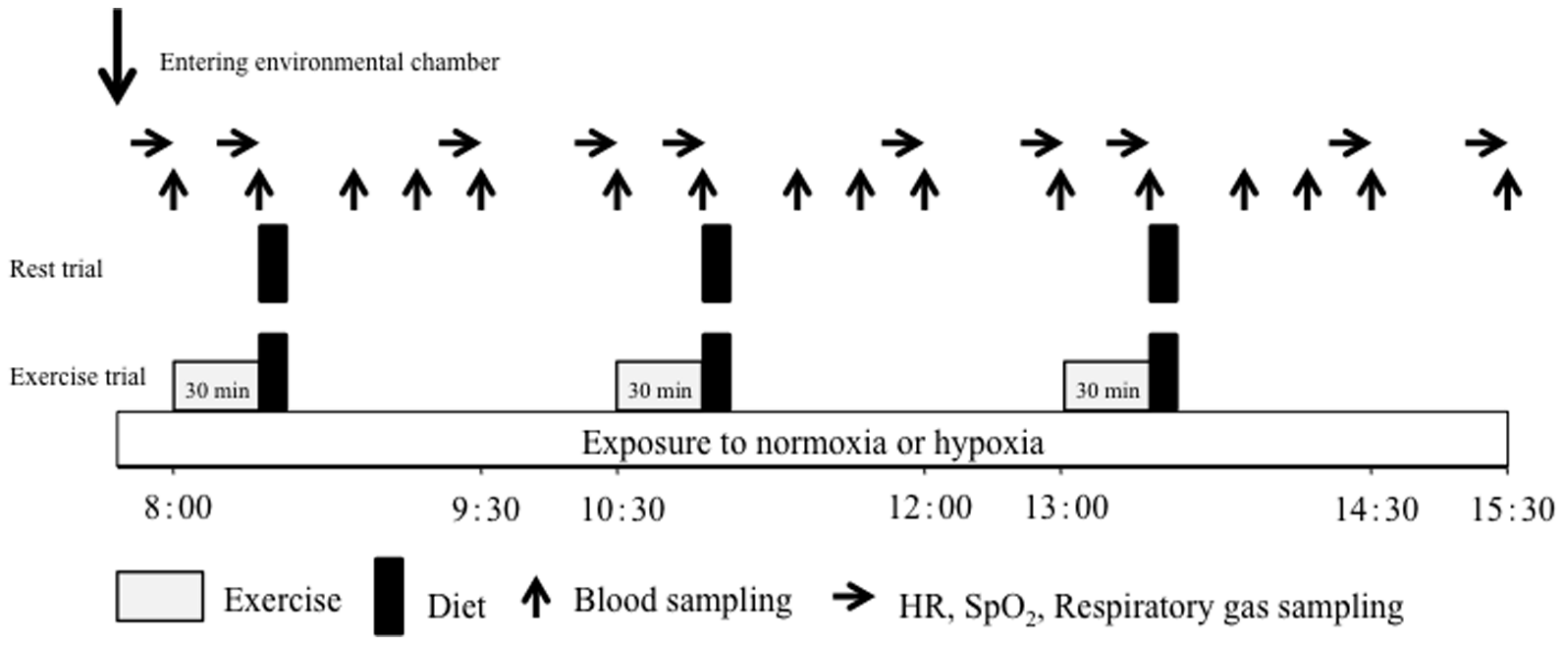
Overview of the study design.

The experimental protocol is shown in Figure 1. The four experimental trials were performed from 8:00 to 15:30 following an overnight fast (at least 10 h). In the rest trials (NOR-Rest and HYP-Rest), subjects rested on a chair, reading books or watching DVDs throughout each trial. In the NOR-Ex and HYP-Ex trials, they conducted three bouts of 30 min pedaling exercise at 60% of VO_2_max at 8:00, 10:30, and 13:00, and rested during the remaining periods in the chamber. Standard meals were provided at 8:30, 11:00, and 13:30, and subjects were instructed to consume the meals within 7 min. The meals consisted of 64% carbohydrate, 10% protein and 26% fat (864 kcal/meal).

### Measurements on experimental trial days

Following an overnight fast, the subjects visited the laboratory at 7:30 in the morning and rested before the first blood collection. After a 30 min rest, a polyethylene catheter was inserted into an antecubital vein and a baseline blood sample was collected. Subsequently, respiratory gas, heart rate (HR), and percutaneous oxygen saturation (SpO_2_) were recorded. During experimental trials, blood samples were collected at baseline and immediately before each meal (at the end of exercise), and at 20, 40, 60, and 120 min after each meal (16 points in total; Fig. 1). Respiratory gas was collected at 10 points (baseline, immediately before, and at 60 and 120 min after each meal) to determine VO_2_, carbon dioxide production (VCO_2_), and ventilatory volume (VE). All respiratory variables were averaged in each 3-min period. The respiratory exchange ratio (RER), determined from the VO_2_ and VCO_2_ measurements, was used to estimate the relative contribution of carbohydrate (%carbohydrate contribution) and fat oxidation (%fat contribution) to total energy production [17]. HR and SpO_2_ were recorded at the same time points as respiratory gas sampling. Rate of perceived exertion (RPE) was monitored at the end of each exercise session.

### Blood analysis

Blood glucose, lactate, serum insulin, free fatty acid (FFA), and glycerol were measured from the whole-blood and serum samples obtained. Serum samples were obtained from blood by centrifugation for 10 min, and were stored at −80°C until analysis. Blood glucose and lactate concentrations were measured immediately after blood collection. Concentrations of blood glucose and lactate were determined using an automatic glucose analyzer (Free Style; Nipro Corporation, Osaka, Japan) and a lactate analyzer (Lactate Pro 2; Arkray Inc. Kyoto, Japan), respectively. The samples for glucose concentrations were analyzed in duplicate. The intraclass coefficient for duplicate measurements in the analysis was 0.99. Serum insulin and FFA concentrations were measured using chemiluminescent enzyme immune assays (Fujirebio Inc., Tokyo, Japan) at a clinical laboratory (SRL Inc., Japan). Serum glycerol concentrations were measured in duplicate using an enzyme-linked immunosorbent assay (Cayman Chemical Company, Ann Arbor, MI, USA). The intra-assay CVs were as follows: 3.3% for serum insulin, 2.2% for serum FFA, and 1.2% for serum glycerol measurements.

### Statistical analysis

Data are expressed as means ± SE. A two-way analysis of variance (ANOVA) with repeated measures was used to test the interaction (trial × time) and main effect (trial, time). When ANOVA revealed a significant interaction or main effect, a Tukey-Kramer test was performed as a *post hoc* analysis to identify differences. For all tests, *P* values <0.05 were considered to indicate statistical significance.

## Results

### SpO_2_ and HR

**Figure 2:**
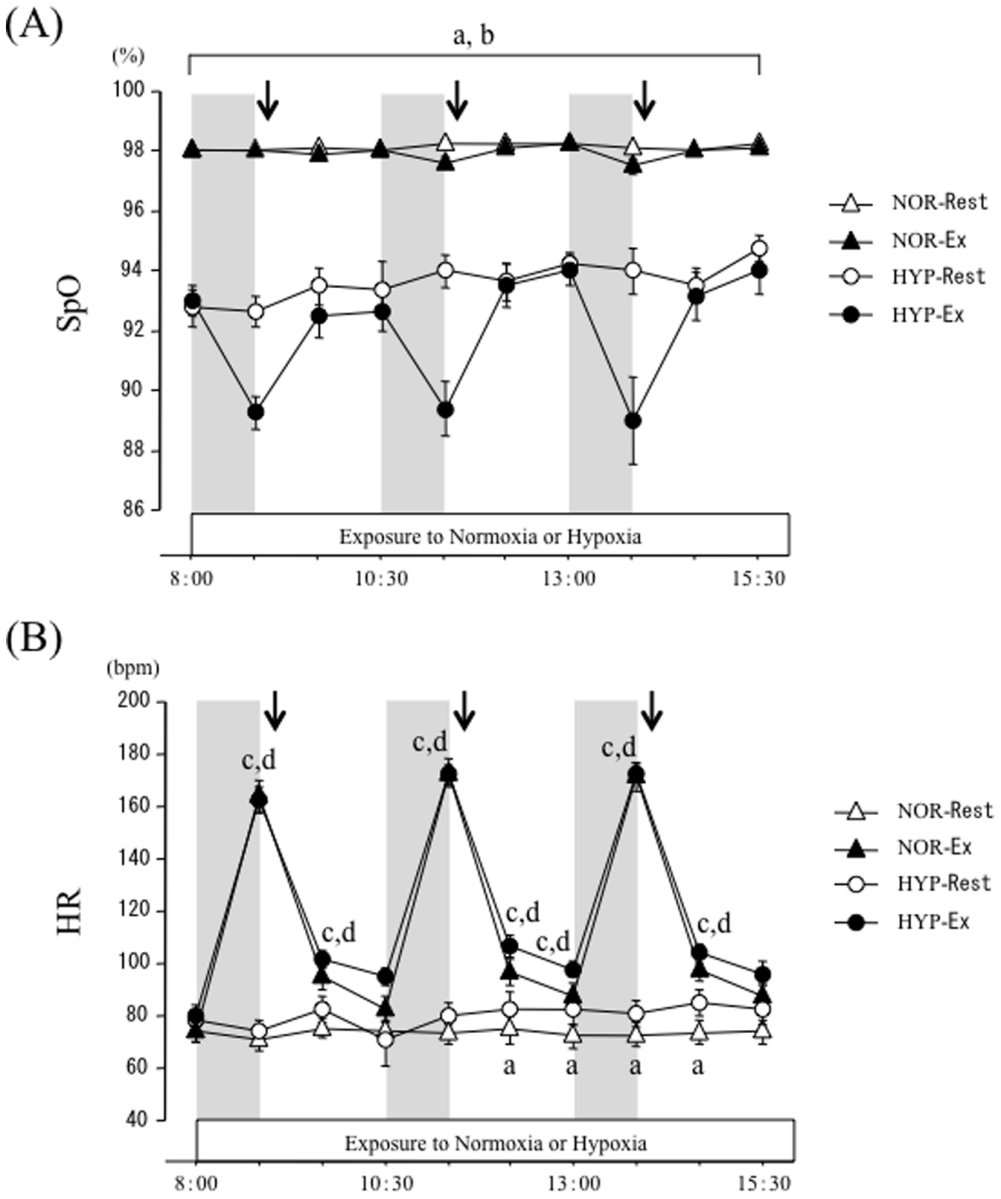
Time-course changes of SpO_2_ (A) and HR (B) over 7.5 h. Each period of exercise is indicated by the shaded boxes. The arrow indicates the time of meal consumption. a; *P*<0.05, NOR-Rest vs. HYP-Rest. b; *P*<0.05, NOR-Ex vs. HYP-Ex. c; *P*<0.05, NOR-Rest vs. NOR-Ex. d; *P*<0.05, HYP-Rest vs. HYP-Ex.

Figure 2 shows the time-course of changes in SpO_2_ and HR over 7.5 h. There were significant interaction (trial × time) and main effects for time and trial (*P*<0.05) for SpO_2_. In NOR-Rest and NOR-Ex trials, SpO_2_ did not change at any time point over the experimental period. The HYP-Rest and HYP-Ex trials showed significantly lower values of SpO_2_ than the NOR-Rest and NOR-Ex trials at all time points (*P*<0.05).

There were significant interaction (trial × time) and main effects for time and trial (*P*<0.05) for HR. HR was significantly higher in the HYP-Rest trial than in the NOR-Rest trial after the second meal. In NOR-Ex and HYP Ex trials, HR was elevated significantly during the exercise period (*P*<0.05). However, HR responses did not differ significantly between the NOR-Ex and HYP-Ex trials over 7.5 h.

### Blood glucose and serum insulin responses

**Figure 3:**
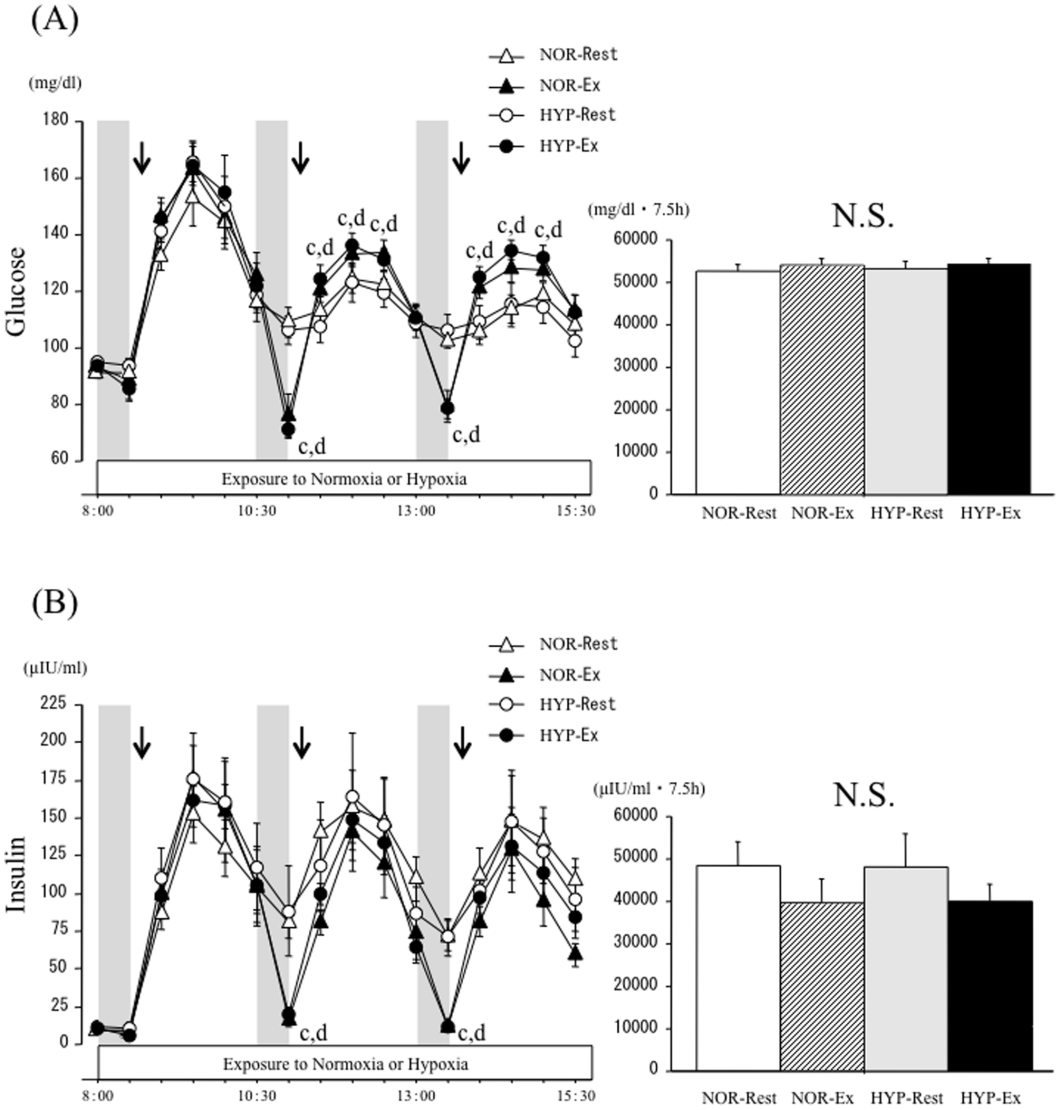
Time-course changes and area under the curve of blood glucose (A) and serum insulin (B) concentrations over 7.5 h. Each period of exercise is indicated by the shaded boxes. The arrow indicates the time of meal consumption. c; *P*<0.05, NOR-Rest vs. NOR-Ex. d; *P*<0.05, HYP-Rest vs. HYP-Ex.

Figure 3 shows the time-course of changes and area under the curve (AUC) over 7.5 h for blood glucose and serum insulin concentrations. There were significant interaction (trial × time) and main effects for time and trial (*P*<0.05) for the blood glucose response. There was no significant difference in fasting glucose concentration at baseline among the four trials. Although blood glucose concentrations increased significantly after each meal in all trials (*P*<0.05), there was no difference in blood glucose concentrations between the NOR-Rest and HYP-Rest trials at any time point. In the NOR-Ex and HYP-Ex trials, although each exercise reduced blood glucose concentrations relative to the values before exercise, blood glucose responses to the second and third meals (postprandial 20, 40, 60 min) were significantly greater than those in the NOR-Rest and HYP-Rest trials (*P*<0.05). When time-course changes in blood glucose concentrations over 7.5 h were compared using area under the curve (AUC), the AUC values did not differ significantly among the four trials.

For serum insulin responses, there were significant interaction (trial × time) and main effects for trial and time (*P*<0.05). Fasting serum insulin concentrations did not differ significantly among the trials. Although serum insulin concentrations increased significantly after each meal in all trials (*P*<0.05), there was no difference in serum insulin concentrations between NOR-Rest and HYP-Rest trials at any time point. In the NOR-Ex and HYP-Ex trials, serum insulin concentrations declined significantly during exercise relative to the values before exercise (*P*<0.05). When time-course changes in serum insulin concentrations over 7.5 h were compared using AUC, the AUC values did not differ significantly among the four trials.

### Serum FFA, glycerol, and blood lactate responses

**Figure 4:**
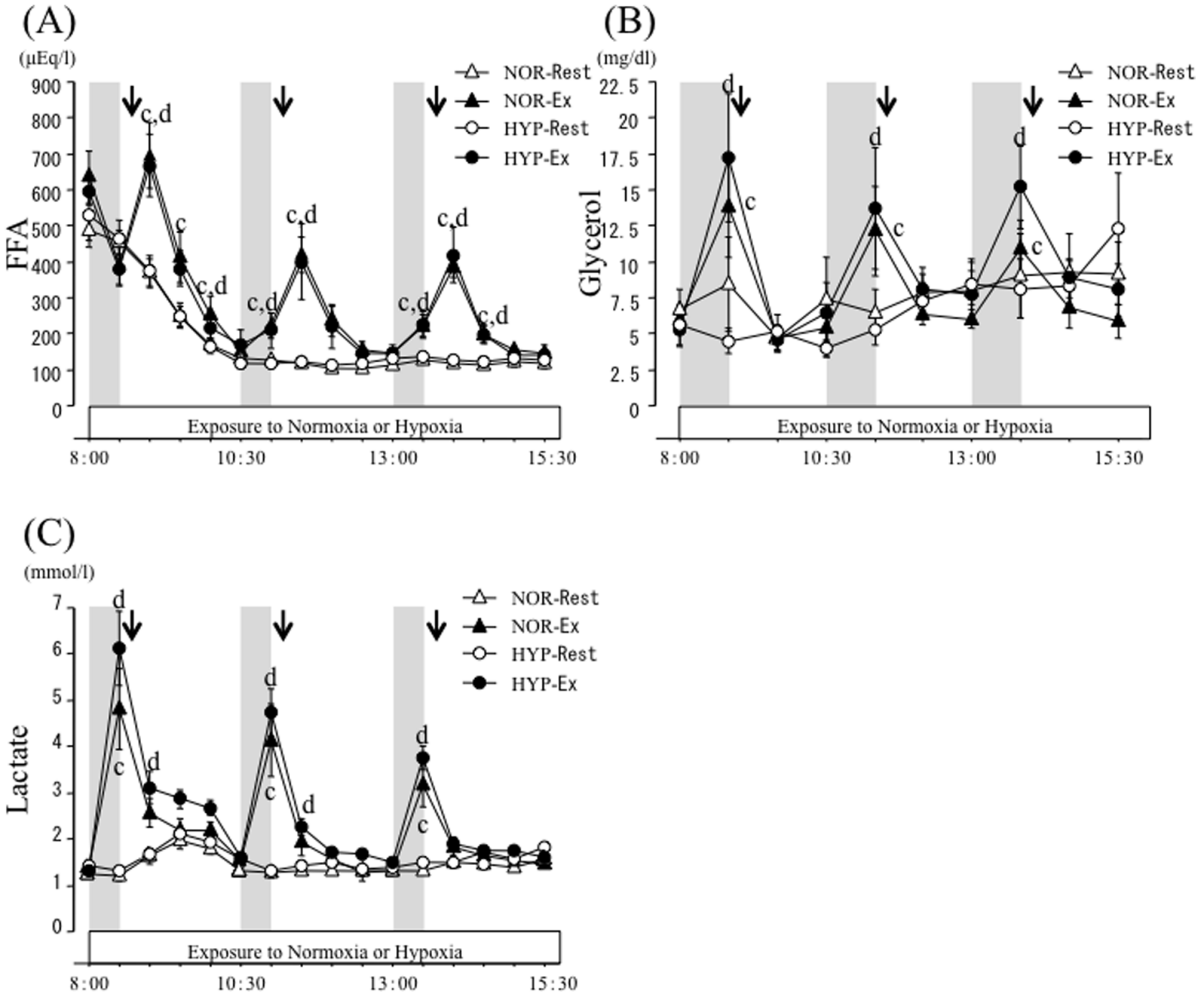
Time-course changes of FFA (A), glycerol (B) and lactate (C) concentrations over 7.5 h. Each period of exercise is indicated by the shaded boxes. The arrow indicates the time of meal consumption. c; *P*<0.05, NOR-Rest vs. NOR-Ex. d; *P*<0.05, HYP-Rest vs. HYP-Ex.

Figure 4 presents the time-course changes in serum FFA, glycerol, and blood lactate concentrations. There were significant interaction (trial × time) and main effects for time and trial (*P*<0.05) in serum FFA concentrations. Fasting serum FFA concentrations did not differ significantly among the four trials. Serum FFA concentrations declined significantly after each meal in all trials (*P*<0.05). In the NOR-Ex and HYP-Ex trials, FFA concentrations increased significantly after each exercise (*P*<0.05). However, FFA responses did not differ significantly between the NOR-Ex and HYP-Ex trials over 7.5 h.

There were significant interaction (trial × time) and main effects for time and trial (*P*<0.05) in serum glycerol concentrations. Fasting blood glycerol concentrations did not differ significantly among the four trials. In the NOR-Rest and HYP-Rest trials, serum glycerol concentrations did not change at any time point over the 7.5 h. In the NOR-Ex and HYP Ex trials, glycerol levels increased significantly during exercise (*P*<0.05). However, glycerol responses did not differ significantly between the NOR-Ex and HYP-Ex trials over the 7.5 h.

For blood lactate responses, there were significant interaction (trial × time) and main effects for time and trial (*P*<0.05). Fasting blood lactate concentrations did not differ significantly among the four trials. Blood lactate concentrations increased significantly after the first meal in NOR-Rest and HYP-Rest (*P*<0.05). In the NOR-Ex and HYP-Ex trials, each exercise period significantly increased blood lactate concentrations relative to baseline values (*P*<0.05). These concentrations returned gradually toward resting levels after consumption of each meal. However, lactate responses did not differ significantly between the NOR-Ex and HYP-Ex trials over the 7.5 h.

### Relative contributions of carbohydrate and fat oxidation

**Figure 5:**
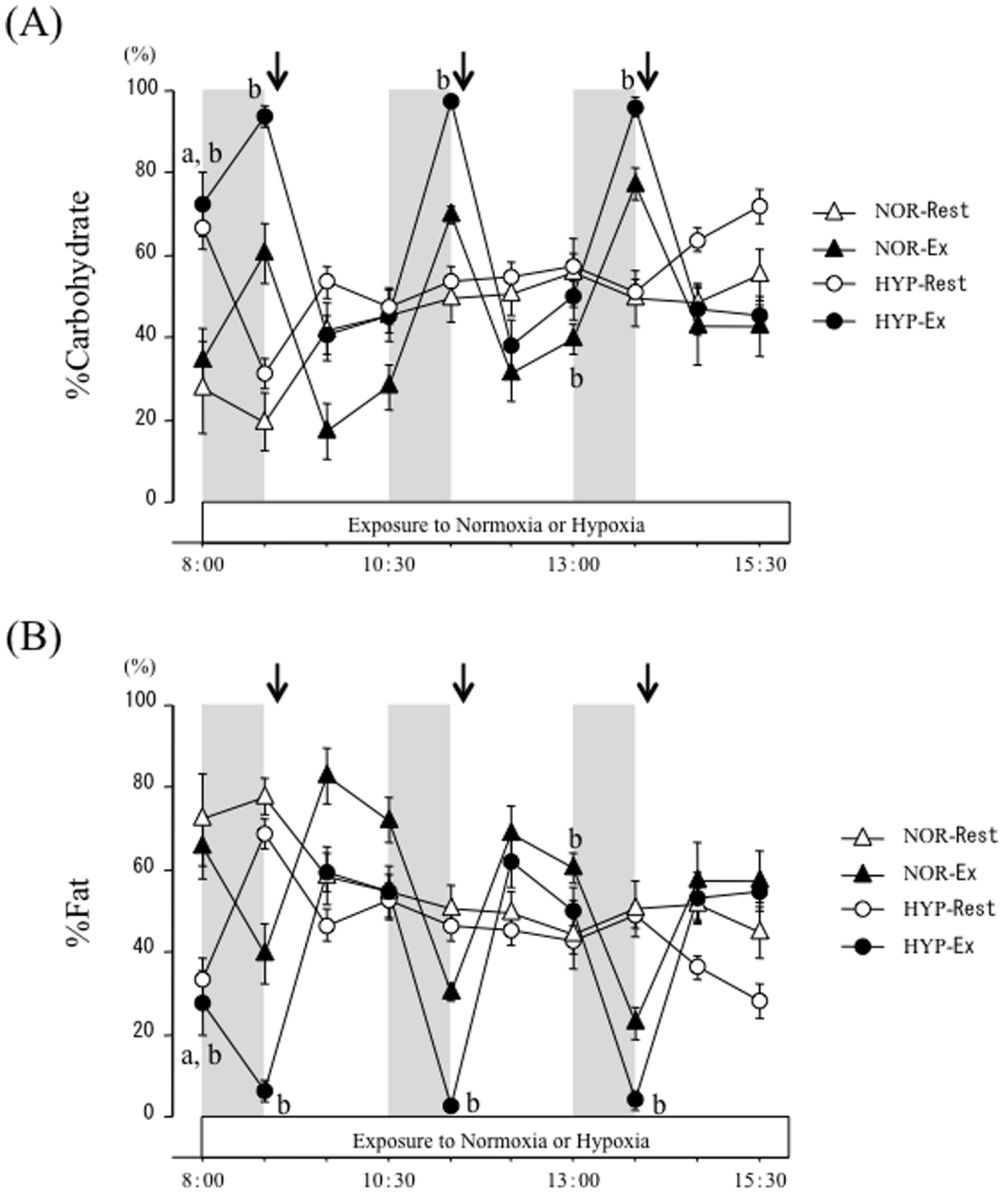
Relative contribution of carbohydrate (A) and fat (B) oxidation over 7.5 h. Each period of exercise is indicated by the shaded boxes. The arrow indicates the time of meal consumption. a; *P*<0.05, NOR-Rest vs. HYP-Rest. b; *P*<0.05, NOR-Ex vs. HYP-Ex.

Time-course changes in relative contributions of carbohydrate (%carbohydrate contribution) and fat oxidation (%fat contribution) to total energy expenditure are shown in Figure 5. The %carbohydrate contribution showed significant interaction (trial × time) and main effects for time and trial (*P*<0.05). At baseline (after entering the chamber), %carbohydrate contribution was significantly higher in the hypoxic trials (HYP-Rest and HYP-EX) than in the normoxic trials (NOR-Rest and NOR-Ex; *P*<0.05). However, no difference between NOR-Rest and HYP-Rest trials was apparent at the remaining points over the experimental period. Although exercise promoted carbohydrate oxidation in the NOR-Ex and HYP-Ex trials, %carbohydrate contribution during exercise was significantly higher in the HYP-Ex trial than in the NOR-Ex trial (*P*<0.05). During the post-exercise period, %carbohydrate contribution remained higher in the HYP-Ex trial than in the NOR-Ex trial, and a significant difference was observed at 120 min after the second exercise period (50.2±5.9% in the HYP-Ex vs. 39.7±3.6% in the NOR-Ex, *P*<0.05).

The time-course changes in %fat contribution showed significant interaction (trial × time) and main effects for time and trial (*P*<0.05). These changes were the opposite of those in %carbohydrate contribution, and values in %fat contribution were significantly lower in the hypoxic trials (HYP-Rest and HYP-Ex) than in the normoxic trials (NOR-Rest and HYP-Ex; *P*<0.05). At baseline, %fat contribution was significantly lower in the hypoxic trials (HYP-Rest and HYP-Ex) than in the normoxic trials (NOR-Rest and NOR-Ex; *P*<0.05). However, there was no significant difference in %fat contribution between NOR-Rest and HYP-Rest trials at the remaining points during the experimental period. The %fat contributions during each exercise period were significantly lower in the HYP-Ex trial than in the NOR-Ex trial (*P*<0.05). Lower levels of %fat contributions in the HYP-Ex trial were maintained during the post-exercise period, and a significant difference was observed at 120 min after the second exercise period (49.8±5.9% in the HYP-Ex vs. 60.3±3.6% in the NOR-Ex; *P*<0.05).

## Discussion

This is the first study to investigate the combined effects of rest and exercise under moderately hypoxic conditions on substrate utilization and endocrine responses in overweight men. Postprandial blood glucose responses in hypoxic trials (HYP-Rest and HYP-Ex) did not differ compared with the normoxic trials (NOR-Rest and NOR-Ex) over 7.5 h. However, the HYP-Ex trial showed significantly higher carbohydrate oxidation than the NOR-Ex trial over the day. The novel finding of the present study was that neither rest alone nor rest and exercise in moderate hypoxia attenuated postprandial glucose responses, despite exercise in moderate hypoxia markedly promoted carbohydrate oxidation over 7.5 h.

AMPK is an intracellular energy-sensing enzyme that promotes blood glucose transport with stimulation, such as exercise or hypoxia [10]. Because skeletal muscle is a major site for postprandial glucose disposal, the AMPK-related glucose transport pathway is considered to play a predominant role in maintaining glucose homeostasis in the whole body [18]–[20]. Thus, we hypothesized that greater glucose uptake via augmented AMPK activation would be observed in the hypoxic trials (HYP-Rest and HYP-Ex) compared with the normoxic trials (NOR-Rest and NOR-Ex). However, the blood glucose response was not significantly different among the trials over 7.5 h. Overweight or obese individuals generally show metabolic abnormality. In fact, AMPK activation has been reported to be impaired in overweight or obese people [21]. Because we selected overweight subjects in the present study, impaired AMPK activation in the present subjects may explain, at least in part, the lack of difference in the postprandial glucose response between the hypoxic and normoxic trials.

As shown in Figure 3, postprandial glucose responses to second and third meals were significantly greater in the exercise trials (NOR-Ex and HYP-Ex) than in the rest trials (NOR-Rest and HYP-Rest). We did not expect these results because exercise has been shown to promote glucose uptake into skeletal muscle [22] and to attenuate postprandial blood glucose responses [23], [24]. However, several previous studies indicated that the postprandial glucose response was aggravated by conducting exercise immediately before a meal [25]–[27]. Aggravated glucose response by prior exercise may be associated with temporal insulin resistance immediately after exercise, characterized by impaired insulin secretion [28] and reduced hepatic glucose uptake [29] in response to the meal consumed. In support of this, insulin responses to the second and third meals in the NOR-Ex and HYP-Ex trials were smaller than in the NOR-Rest and HYP-Rest trials. In future studies, optimal timing of exercise to prevent increased postprandial glucose responses should be examined.

A unique component of the present study was the comparison of time-course changes in substrate utilization patterns between normoxic and hypoxic conditions over 7.5 h in overweight men. In baseline data, evaluated after entering the environmental chamber, RER was significantly higher in the hypoxic trials than in the normoxic trials (*P*<0.05, data not shown). It is well known that acute hypoxic exposure induces hyperventilation [30], [31]. This may cause an overestimation of CO_2_ production [32]–[34] and affect the validity of calculating RER due to the elevated CO_2_. However, there was no significant difference in VE at baseline between the normoxic trials and hypoxic trials. Thus, it seems unlikely that hypoxia-induced hyperventilation affected the results of the present study. However, the higher levels of %carbohydrate contribution at baseline in hypoxic trials were temporary responses because there was no significant difference in %carbohydrate contribution between NOR-Rest and HYP-Rest trials at the remaining measurement points. We think that moderate hypoxic exposure alone did not have a strong impact on metabolic or endocrine responses.

The relative contribution of carbohydrate oxidation (%carbohydrate contribution) during the exercise and post-exercise periods was significantly higher in the HYP-Ex trial than in the NOR-Ex trial, consistent with several previous reports. Peronnet et al., 2006 [8] and Katayama et al., 2010 [35] reported that carbohydrate oxidation during prolonged exercise was significantly higher in hypoxic conditions than in normoxic conditions. Brooks et al., 1991 [6], 1992 [36] and Roberts et al., 1996a [37], b [38] suggested that enhanced reliance on plasma glucose oxidation for energy production under hypoxic conditions leads to the promotion of carbohydrate oxidation during exercise. Data from the present study indicate that “moderate hypoxia” and “exercise” have synergistic effects in promoting carbohydrate oxidation during exercise and post-exercise periods. However, blood glucose and insulin responses did not differ between NOR-Ex and HYP-Ex trials over 7.5 h. Additionally, blood lactate concentrations during exercise did not differ significantly between the NOR-Ex and HYP-Ex trials. Thus, increased carbohydrate oxidation in the HYP-Ex trial was apparently not sufficient to affect blood variables regarding glucose metabolism.

The relative contributions of fat oxidation (%fat contribution) during exercise and the post-exercise period were significantly lower in the HYP-Ex trial than in the NOR-Ex trial. The reduced fat oxidation under hypoxic conditions agrees with a previous study that reported impaired adipose tissue lipolysis after prolonged exposure to hypoxia [39]. However, in the present study, there was no significant difference in serum FFA or glycerol concentrations during exercise between the NOR-Ex and HYP-Ex trials, suggesting that exercise-induced adipose tissue lipolysis was not attenuated by moderate hypoxic exposure. An inconsistent result between fat oxidation and lipolysis has been reported previously. Ohkawara et al., 2013 [40] indicated that an increased meal frequency decreased FFA concentrations over a day, in contrast to fat oxidation, which was unchanged.

We understand that some limitations should be carefully considered for interpretation of the present study. First, subjects in the present study were overweight men, but they did not have any metabolic disorder, which may affect the present results. Further studies using severely obese people or people with type 2 diabetes are necessary to extend our understanding. Second, substrate levels in blood do not correspond correctly to substrate utilization because substrate levels such as glucose, lactate or FFA are influenced by the rates of appearance and disappearance. This will partly explain for lack of attenuations of glucose and insulin responses in hypoxia, in despite of augmented carbohydrate oxidation.

## Conclusion

Neither rest alone nor rest and exercise under moderately hypoxic conditions attenuated postprandial glucose responses. Additionally, rest alone under moderately hypoxic condition did not affect the substrate oxidation pattern markedly. However, carbohydrate oxidation was enhanced significantly over 7.5 h when three bouts of submaximal exercise were incorporated under moderately hypoxic conditions.
